# Trajectories of patient-reported outcomes among diverse cancer patients in ambulatory oncology clinics

**DOI:** 10.1007/s11764-026-02050-0

**Published:** 2026-06-04

**Authors:** Akina Natori, Blanca Silvia Noriega Esquives, Vandana D. Sookdeo, Rui Gong, Jessica MacIntyre, Patricia I. Moreno, Carmen Calfa, Tracy E. Crane, Matthew Schlumbrecht, Frank J. Penedo

**Affiliations:** 1 Division of Medical Oncology, Department of Medicine, University of Miami Miller School of Medicine, Miami, FL USA; 2 Sylvester Comprehensive Cancer Center, University of Miami, Miami, FL USA; 3 Department of Public Health Sciences, University of Miami Miller School of Medicine, Miami, FL USA; 4 Division of Gynecologic Oncology, Department of Obstetrics and Gynecology, University of Miami Miller School of Medicine, Miami, FL USA; 5 Department of Medicine, University of Miami Miller School of Medicine, 1120 NW 14 Street, Miami, FL 33136 USA; 6 Department of Psychology, University of Miami, Coral Gables, USA

**Keywords:** Cancer survivors, Patient-reported outcome measures, Health-related quality of life, Longitudinal studies

## Abstract

**Purpose:**

Tracking patient-reported outcomes (PROs) offers critical insights into cancer survivors’ symptoms and health-related quality of life (HRQOL). This study evaluated longitudinal trajectories of PROs across cancer types and identified factors associated with symptom burden and HRQOL.

**Methods:**

We conducted a retrospective longitudinal observational study of 6939 cancer survivors (breast, prostate, lung, head and neck (H&N), gastrointestinal (GI), gynecological (GYN), and hematological malignancies) in ambulatory oncology clinics. All cancer survivors completed electronic health record–integrated PRO assessments measuring anxiety, depression, pain interference, fatigue, and physical function, and HRQOL. Linear mixed-effect models evaluated difference in PRO level and change over time by cancer types, adjusting for sociodemographic and clinical covariates.

**Results:**

Meaningful differences in overall symptom burden were observed across cancer types. Survivors with GYN cancers reported consistently higher anxiety, pain interference, fatigue, and poorer physical function, whereas prostate cancer survivors reported lowest symptom burden and highest HRQOL. Hematologic malignancy survivors improved in physical function over time compared to prostate cancer survivors. Chemotherapy and surgery were associated with greater pain interference, fatigue, reduced physical function, and lower HRQOL. Greater comorbidity burden and being unpartnered were associated with worse PRO.

**Conclusions:**

PRO trajectories varied by cancer types and patient characteristics, underscoring the need for tailored, cancer-specific survivorship care strategies.

**Implications for Cancer Survivors:**

Routine, EHR-integrated PRO monitoring can identify survivors at risk for persistent symptom burden and support timely, individualized interventions to improve long-term HRQOL.

**Supplementary Information:**

The online version contains supplementary material available at https://doi.org/10.1007/s11764-026-02050-0.

## Introduction

Patient-reported outcomes (PROs) capture patients’ perceptions of their health status, quality of life, and the impact of disease and treatment on their daily lives, without interpretation by healthcare providers [1]. PROs encompass a broad spectrum of self-reported information, including physical symptoms, emotional well-being, functional status, and health-related quality of life (HRQOL) [2]. In clinical research and practice, PROs provide invaluable insights into patients’ experiences, enhancing the understanding of treatment efficacy, guiding clinical decision-making, and promoting patient-centered care [3, 4]. Over the past few decades, PROs have been increasingly integrated into clinical trials to assess the effectiveness and tolerability of investigational therapies. More recently, the implementation of PROs in routine clinical workflows, particularly in chronic disease management such as cancer, has gained momentum. PROs are now frequently used in quality improvement initiatives, allowing for continuous symptom monitoring and identifying areas where interventions may improve outcomes [5]. 

Beyond symptom assessment, PROs, particularly HRQOL measures, are emerging as important prognostic and predictive tools in oncology [6]. Numerous studies have demonstrated that better HRQOL is often associated with better treatment responses and improved survival outcomes in patients with cancer [7–10]. For example, a large-scale analysis of the data from 30 randomized controlled trials across 11 cancer types conducted by the European Organization for Research and Treatment of Cancer (EORTC) found that specific HRQOL domains provided independent prognostic information after adjusting for clinical and sociodemographic variables [11, 12]. Similarly, research from a population-based cancer registry in the Netherlands found a strong association between HRQOL summary scores and overall survival across 12 different cancer types [13].

Despite these findings, the prognostic value of different PRO domains can vary by cancer type, and most research to date has been cross-sectional, limiting understanding of longitudinal trajectories of PROs [8]. Understanding how PROs change over time, particularly across different types of cancer, is crucial for identifying vulnerable patient populations and key domains for targeted interventions. While some longitudinal studies have explored PRO trajectories in specific cancer types or treatment contexts, comprehensive analyses that span multiple cancer types are scarce. Such comparisons are vital for identifying patterns and differences in PRO trajectories that could inform personalized care strategies. This observational longitudinal study aims to identify the trajectories of various PRO domains across multiple cancer types and examine the associations between these trajectories and patient characteristics.

## Methods

### Patient population

The Sylvester Comprehensive Cancer Center (SCCC) implemented the My Wellness Check (*MWC*) program, a routine electronic health record (EHR)-based PRO and needs screening system, across ambulatory oncology clinics. The *MWC* questionnaire is linked to patients’ scheduled ambulatory oncology clinic visits. If a patient has completed the questionnaire within the past 30 days, it is not re-assigned; otherwise, it is administered again with the next eligible visit. As a result, the timing of PRO assessments is not based on fixed intervals. Patients receive the *MWC* questionnaire via the patient portal. The *MWC* questionnaire includes five PROMIS® computerized adaptive tests (CATs)—anxiety, depression, pain interference, fatigue, and physical function—to assess common cancer-related symptoms [14–18]. It also includes the Functional Assessment of Cancer Therapy-General (7-item version; FACT-G7) [19] to evaluate HRQOL. Participation in the *MWC* questionnaire is optional. Detailed program descriptions have been published previously [20, 21].

Among 13,403 patients who were assigned to the *MWC* questionnaire, the patients who met the following inclusion criteria were included in this study: (1) age 18 years or older, (2) a diagnosis of cancer at any site or stage, within 5 years of the first MWC assessment, and (3) completion of at least one *MWC* questionnaire between May 2022 and November 2023. Due to the limited number of patients with other cancer types, only the seven most frequent cancer types, including breast, gynecological (GYN), gastrointestinal (GI), head and neck, hematologic, lung, and prostate malignancies, were included. The final sample included 6939 cancer survivors. The institutional review board approved the study protocol at the University of Miami (ePROST#20200984). Informed consent was waived because this study retrospectively used the clinical data that was routinely collected as a standard of care.

### Data collection

*MWC* questionnaire scores are automatically calculated and stored in the EHR. For this study, all relevant scores were retrospectively collected from the electronic data warehouse (EDW). The main outcomes of interest were HRQOL, as assessed by the FACT-G7, and symptom burden, as assessed by the five PROMIS®-CAT domains in the *MWC* program [14–19]. PROMIS® measures use a T-score metric, where 50 represents the mean of a relevant reference population, and 10 represents the standard deviation. Higher scores indicate an increase in the measured construct (e.g., greater depression, better physical function). In addition, a change of 2 to 6 T-score points is widely accepted as a clinically relevant difference, applicable to both within-person changes and comparisons between distinct groups [22]. FACT-G7 score ranges from 0 to 28, which higher scores indicate better HRQOL [23]. Patient demographics, including age, sex, race/ethnicity, marital status, and insurance status, and clinical characteristics at the time of the first assessment, including cancer site, time since cancer diagnosis, cancer stage, current treatment type, and comorbidities, were also collected from the EDW. Charlson Comorbidity Index was calculated to assess the severity of comorbidity [24]. Receipt of treatment was defined as a treatment within 30 days before each assessment. Metastatic disease was defined as stage IV cancer or the presence of ICD-10 codes for secondary malignancy (C77–C79). For lymphoma, staging followed the Lugano classification, and stage IV disease, indicating disseminated extranodal involvement was categorized as metastatic, consistent with prior literature and clinical convention. All leukemia cases were classified as having metastatic disease for this analysis because leukemia is an inherently systemic hematologic malignancy and is considered disseminated at diagnosis in standard registry staging [25].

### Statistical data analysis

Descriptive statistics were calculated for characteristics at the first assessment using chi-square tests and analysis of variance (ANOVA) tests where appropriate. Linear mixed-effects models were employed to assess the impact of time and cancer type on PROMIS and FACT-G7 scores. These models are particularly useful for analyzing repeated measures data, where assessments (or time points) are nested within individuals [26]. At level 1, time was treated as a continuous fixed effect, representing the number of months between the initial cancer diagnosis and the date of each assessment. Person-level predictors (level 2) included cancer site (reference category = prostate cancer), age at the first assessment, marital status (partnered: yes/no), race/ethnicity (reference: non-Hispanic White), insurance status (insured: yes/no), the presence of metastatic disease (yes/no), Charlson Comorbidity Index, and treatments performed at each assessment point (radiotherapy, chemotherapy, surgery, immunotherapy, and hormonal therapy, coded as yes/no). Additionally, we included an interaction term for the time of assessment by cancer type. Multicollinearity among predictors was assessed using the variance inflation factor (VIF) and Cramér’s *V*, confirming that the associations between predictors did not compromise model stability. Sex was not included in the models to avoid multicollinearity arising from its near-perfect correlation with site-specific cancers (e.g., prostate, breast, and female genital system). Continuous variables (i.e., age and comorbidities) were grand mean-centered before modeling to facilitate the clinical interpretation of the intercept and to reduce potential multicollinearity between predictors. We added the random effects of time and intercept to the model to account for variability in the number of assessments and their intervals, especially considering that the MWC questionnaire is linked to patient clinic visits. This allows the model to estimate unique trajectories for each participant, accounting for the fact that patients may experience changes in PRO scores at different rates. An unstructured covariance matrix was utilized to allow for correlation between the random intercepts and random slopes, ensuring the model captured the relationship between baseline scores and subsequent rates of change. Model fit was assessed using the Akaike Information Criterion (AIC) and Bayesian Information Criterion (BIC). Likelihood ratio tests confirmed that the inclusion of a random slope for time significantly improved model fit for all PRO outcomes (*p* < 0.001), and thus, it was retained in the final models. Unstandardized regression coefficients (Bs), standard errors (SE), and *p* values were assessed. Fixed-effect significance was determined using type III tests. Due to the multiple comparisons, a Bonferroni-adjusted significance level of 0.0083 was calculated to account for the increased possibility of type-I error. The predicted means of individual cancer types were plotted graphically across time (0–60 months since cancer diagnosis), assuming random intercepts and slopes and a reference patient profile (age: 63.3 years, time: 0 months, non-Hispanic White, unpartnered, comorbidities: 5.8, prostate cancer, non-metastatic disease, not receiving treatment at each assessment). Data analysis was performed using SAS v9.4 (SAS Institute Inc., Cary, NC).

## Results

### Patient and clinical characteristics

A total of 9331 patients completed the *MWC* questionnaire at least once during the study period. After excluding patients diagnosed with cancer more than 5 years prior to their initial assessment, 6939 patients were included in the analysis. The median number of assessments per patient was 2 (range: 1–12). About one third of the sample completed one questionnaire (*n* = 2258 (32.5%)). Over half the sample (*n* = 3585; 51.7%) completed two to four, and 15.8% (*n* = 1096) completed five or more questionnaires. The median time from diagnosis to the initial assessment was 12 months (interquartile range: 2.9–29.5).

The mean age at first assessment was 63.3 years (SD = 13.1). Of the included patients, 3005 (43.3%) were female, and 3015 (43.5%) identified as Hispanic. Most patients were insured (*n* = 6785, 97.8%) and partnered (*n* = 4346, 62.6%). Hematologic malignancies were the most common cancer type (*n* = 1802, 26%), followed by prostate (*n* = 1428, 20.6%) and GI (*n* = 1418, 20.4%). The average time since diagnosis was 18 months (SD = 17.1), and nearly one third (*n* = 2054, 29.6%) had metastatic disease at the first assessment. Additional characteristics, including cancer-specific breakdowns, are summarized in Table 1. PRO score categories at first assessment indicate that patients with prostate cancers consistently reported the highest percentages of “normal” levels for anxiety, depression, and fatigue. Conversely, GYN and lung cancer patients exhibited a higher prevalence of moderate or severe symptoms across most PROs (Table 2). Participants who completed multiple assessments were more likely to be female and non-Hispanic Black, had higher Charlson Comorbidity Index scores, and were more frequently receiving treatments, including chemotherapy, immunotherapy, or hormonal therapy compared to those who completed only one assessment (Online Resource Table S1). Conversely, single-assessment participants were more likely to have metastatic disease, higher pain interference, and a more recent cancer diagnosis (Online Resource Table S1).

**Table 1:** Patient characteristics at baseline

	**Total** (*N* = 6939)		**Breast** (*N* = 518)		**GYN** (*N* = 425)		**GI** (*N* = 1418)		**Head and neck** (*N* = 696)		**Hematologic** (*N* = 1802)		**Lung** (*N* = 652)		**Prostate** (*N* = 1428)		***p* value**^a^
	**Mean or *N***	**SD or %**	**Mean or *N***	**SD or %**	**Mean or *N***	**SD or %**	**Mean or *N***	**SD or %**	**Mean or *N***	**SD or %**	**Mean or *N***	**SD or %**	**Mean or *N***	**SD or %**	**Mean or *N***	**SD or %**	
**Age at baseline**	63.3	13.1	60.1	12.9	59.8	13.1	62.4	12.6	64.7	11.5	61.4	15	67.3	11.6	66.2	11.1	** <.0001**
**Female**	3005	43.3	518	100	425	100	648	45.7	199	28.6	852	47.3	363	55.7	0	0	** <.0001**
**Partnered**	4346	62.6	272	52.5	210	49.4	850	59.9	433	62.2	1115	61.9	387	59.4	1079	75.6	** <.0001**
**Insured**	6785	97.8	508	98.1	416	97.9	1383	97.5	682	98	1763	97.8	640	98.2	1393	97.5	0.963
**Race/ethnicity**																	
Non-Hispanic White	2765	39.8	189	36.5	141	33.2	514	36.2	342	49.1	734	40.7	282	43.3	563	39.4	** <.0001**
Non-Hispanic Black	224	3.23	30	5.79	19	4.47	39	2.75	17	2.44	67	3.72	14	2.15	38	2.66	**0.003**
Non-Hispanic Other	591	8.52	42	8.11	54	12.7	110	7.76	43	6.18	149	8.27	38	5.83	155	10.9	** <.0001**
Hispanic	3015	43.5	240	46.3	191	44.9	683	48.2	260	37.4	761	42.2	287	44	593	41.5	** <.0001**
Missing	344	4.96	17	3.28	20	4.71	72	5.08	34	4.89	91	5.05	31	4.75	79	5.53	
**Charlson Comorbidity Index**	5.83	3.62	8.31	2.53	6.36	3.58	7.51	3.51	6.83	3.42	3.87	2.84	7.58	3.38	4.27	3.15	** <.0001**
**Cancer site**																	
Breast	518	7.46	518	100													
Female genital system	425	6.12			425	100											
Gastrointestinal	1418	20.44					1418	100									
Head and neck	696	10.03							696	100							
Hematologic	1802	25.97									1802	100					
Lung	652	9.40											652	100			
Prostate	1428	20.58													1428	100	
**Months since diagnosis**	17.99	17.1	20.3	17.3	16.9	16.6	15.2	16.1	20	18	20.4	17.6	15	15.9	17.6	17	** <.0001**
**Metastatic disease**	2988	43.1	422	81.5	142	33.4	627	44.2	277	39.8	943	52.3	298	45.7	279	19.5	** <.0001**
**Treatment type**																	
Chemotherapy	1692	24.4	181	34.9	105	24.7	481	33.9	80	11.5	611	33.9	165	25.3	69	4.83	** <.0001**
Immunotherapy	321	4.63	15	2.9	28	6.59	107	7.55	21	3.02	22	1.22	120	18.4	8	0.56	** <.0001**
Hormonal therapy	434	6.25	138	26.6	12	2.82	18	1.27	5	0.72	26	1.44	11	1.69	224	15.7	** <.0001**
Surgery	576	8.3	31	5.98	25	5.88	64	4.51	102	14.7	18	1	75	11.5	261	18.3	** <.0001**
Radiation	123	1.77	10	1.93	3	0.71	26	1.83	31	4.45	14	0.78	21	3.22	18	1.26	** <.0001**
**Patient-reported outcome**																	
Anxiety	51	9.99	53.3	9.73	53	10.2	51.2	10.1	51	10.4	51	9.83	52.5	9.79	48.8	9.54	** <.0001**
Depression	46.4	9.58	48.3	9.29	47.7	9.96	46.7	9.54	46.1	9.75	46.3	9.65	47.5	9.99	44.5	8.95	** <.0001**
Pain interference	51.4	10.7	52.6	10.8	53.4	11	51.8	11.1	51.5	11.2	50.9	10.4	52.6	11.1	49.9	9.8	** <.0001**
Fatigue	48.4	11.4	50.7	11.1	49.9	11.2	49.2	11.6	47.9	12	49.2	11.2	49.6	11.4	44.9	10.3	** <.0001**
Physical function	44.9	11.3	43.7	10.1	43.9	11	43.6	11.2	45.8	11.9	45.1	11.5	42.2	10.7	47.6	11.3	** <.0001**
Health-related quality of life	19.3	5.76	18.4	5.62	18.6	6.02	18.5	6.02	19.1	6.17	19.3	5.6	18.5	6.05	21	4.88	** <.0001**

*GI*, gastrointestinal cancer; *GYN*, gynecological cancer; *HRQOL*, health-related quality of life; *SD*, standard deviation

^a^Statistically significant *p* values are indicated in bold. (Bonferroni-adjusted *p* <.0083)

**Table 2:** Baseline patient-reported outcome severity levels by cancer type

	**Breast** (*N* = 518)	**GYN** (*N* = 425)	**GI** (*N* = 1418)	**Head and neck** (*N* = 696)	**Hematologic** (*N* = 1802)	**Lung** (*N* = 652)	**Prostate** (*N* = 1428)	***p* value**^a^
**Anxiety**								** <.0001**
Normal	278 (53.7%)	225 (52.9%)	848 (59.8%)	426 (61.2%)	1075 (59.6%)	367 (56.3%)	980 (68.6%)	
Mild	87 (16.8%)	66 (15.5%)	206 (14.5%)	96 (13.8%)	301 (16.7%)	103 (15.8%)	172 (12.0%)	
Moderate	118 (22.8%)	101 (23.8%)	274 (19.3%)	120 (17.2%)	296 (16.4%)	138 (21.2%)	186 (13.0%)	
Severe	17 (3.3%)	18 (4.2%)	36 (2.5%)	21 (3.0%)	34 (1.9%)	17 (2.6%)	11 (0.8%)	
Missing	18 (3.5%)	15 (3.5%)	54 (3.8%)	33 (4.7%)	96 (5.3%)	27 (4.1%)	79 (5.5%)	
**Depression**								** <.0001**
Normal	325 (62.7%)	278 (65.4%)	973 (68.6%)	491 (70.6%)	1244 (69.0%)	427 (65.5%)	1064 (74.5%)	
Mild	74 (14.3%)	56 (13.2%)	153 (10.8%)	65 (9.3%)	187 (10.4%)	81 (12.4%)	132 (9.2%)	
Moderate	48 (9.3%)	34 (8.0%)	105 (7.4%)	59 (8.5%)	111 (6.2%)	55 (8.4%)	62 (4.3%)	
Severe	3 (0.6%)	6 (1.4%)	10 (0.7%)	2 (0.3%)	16 (0.9%)	9 (1.4%)	2 (0.1%)	
Missing	68 (13.1%)	51 (12.0%)	177 (12.5%)	79 (11.3%)	244 (13.5%)	80 (12.3%)	168 (11.8%)	
**Pain interference**								** <.0001**
Normal	244 (47.1%)	203 (47.8%)	713 (50.3%)	358 (51.4%)	970 (53.8%)	308 (47.2%)	826 (57.8%)	
Mild	91 (17.6%)	70 (16.5%)	217 (15.3%)	83 (11.9%)	274 (15.2%)	109 (16.7%)	228 (16.0%)	
Moderate	98 (18.9%)	71 (16.7%)	250 (17.6%)	132 (19.0%)	293 (16.3)	121 (18.6%)	197 (13.8%)	
Severe	24 (4.6%)	34 (8.0%)	82 (5.8%)	40 (5.8%)	60 (3.3%)	39 (6.0%)	27 (1.9%)	
Missing	61 (11.8%)	47 (11.1%)	156 (11.0%)	83 (11.9%)	205 (11.4%)	75 (11.5%)	150 (10.5%)	
**Fatigue**								** <.0001**
Normal	278 (53.7%)	247 (58.1%)	787 (55.5%)	425 (61.1%)	1041 (57.8%)	372 (57.1%)	1012 (70.9%)	
Mild	67 (12.9%)	51 (12.0%)	192 (13.5%)	64 (9.2%)	202 (11.2%)	79 (12.1%)	120 (8.4%)	
Moderate	68 (13.1%)	50 (11.8%)	181 (12.8%)	76 (10.9%)	221 (12.3%)	82 (12.6%)	83 (5.8%)	
Severe	18 (3.5%)	15 (3.5%)	46 (3.2%)	27 (3.9%)	49 (2.7%)	24 (3.7%)	12 (0.8%)	
Missing	87 (16.8%)	62 (14.6%)	212 (15.0%)	104 (14.9%)	289 (16.0%)	95 (14.6%)	201 (14.1%)	
**Physical function**								** <.0001**
Normal	50 (9.7%)	48 (11.3%)	157 (11.1%)	116 (16.7%)	275 (15.3%)	53 (8.1%)	275 (19.3%)	
Mild	229 (44.2%)	180 (42.4%)	572 (40.3%)	275 (39.5%)	714 (39.6%)	258 (39.6%)	592 (41.5%)	
Moderate	118 (22.8%)	94 (22.1%)	323 (22.8%)	139 (20.0%)	385 (21.4%)	172 (26.4%)	218 (15.3%)	
Severe	47 (9.1%)	46 (10.8%)	146 (10.3%)	63 (9.1%)	149 (8.3%)	74 (11.4%)	83 (5.8%)	
Missing	74 (14.3%)	57 (13.4%)	220 (15.5%)	103 (14.8%)	279 (15.5%)	95 (14.6%)	260 (18.2%)	
**HRQOL**								** <.0001**
Low	135 (26.1%)	114 (26.8%)	390 (27.5%)	180 (25.9%)	437 (24.3%)	179 (27.5%)	209 (14.6%)	
Normal/high	259 (50.0%)	222 (52.2%)	701 (49.4%)	377 (54.2%)	967 (53.7%)	315 (48.3%)	880 (61.6%)	
Missing	124 (23.9%)	89 (20.9%)	327 (23.1%)	139 (20.0%)	398 (22.1%)	158 (24.2%)	339 (23.7%)	

*GI*, gastrointestinal cancer; *GYN*, gynecological cancer; *HRQOL*, health-related quality of life

^a^Pearson’s chi-squared test. Statistically significant p values are indicated in bold.(Bonferroni-adjusted p < .0083)

### Trajectories and predicted means of each patient-reported outcome

**Fig. 1:**
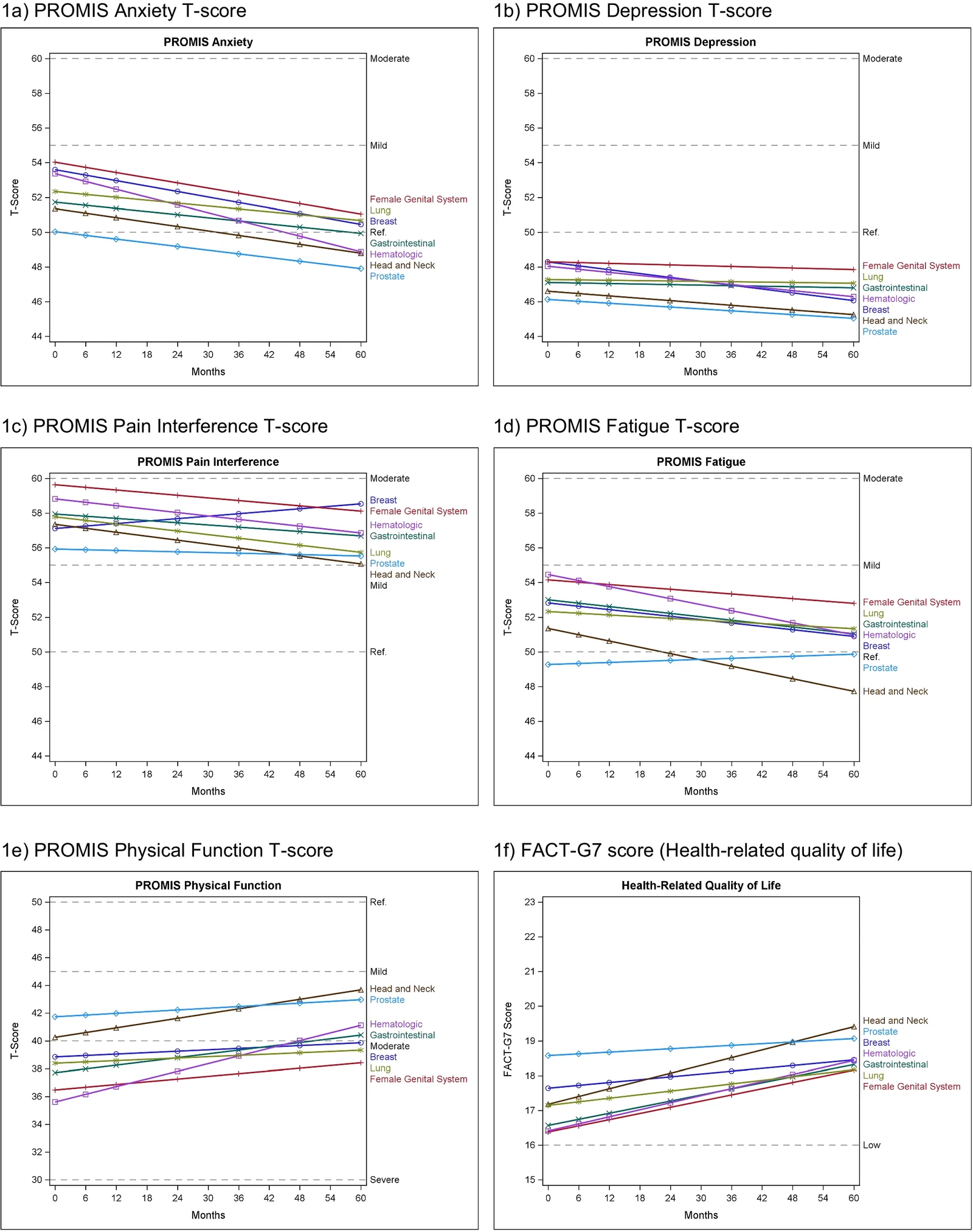
**a**–**f** Trajectory of PRO by domain

The final models and results are summarized in Table 3, and the predicted means of each PRO across time by cancer type are shown in Online Resource Table S2. Figure 1 illustrates the trajectory of each PRO by cancer type.

**Table 3:** Factors associated with patient-reported outcomes

	**Anxiety**		**Depression**		**Pain interference**		**Fatigue**		**Physical function**		**HRQOL**	
	**Estimate**	**SE**	**Estimate**	**SE**	**Estimate**	**SE**	**Estimate**	**SE**	**Estimate**	**SE**	**Estimate**	**SE**
**Intercept**	48.99*	1.08	45.69*	1.06	44.99*	1.13	42.87*	1.24	65.91*	1.18	21.92*	0.65
**Time**	−0.04	0.01	−0.02	0.01	−0.01	0.01	0.01	0.02	0.02	0.01	0.01	0.01
**Age at baseline**	−0.01	0.01	0.01	0.01	0.04*	0.01	0.03	0.01	−0.22*	0.01	−0.01	0.01
**Partnered vs. unpartnered**	−1.06*	0.24	−1.48*	0.24	−0.99*	0.25	−1.07*	0.28	1.05*	0.26	0.72*	0.14
**Insured vs. uninsured**	−0.52	0.78	−1.33	0.78	−0.41	0.83	−0.54	0.90	0.03	0.86	0.39	0.48
**Charlson Comorbidity Index**	0.27*	0.04	0.30*	0.04	0.40*	0.04	0.47*	0.04	−0.63*	0.04	−0.28*	0.02
**Metastatic disease**	0.35	0.27	0.32	0.26	1.04*	0.28	1.02*	0.31	−0.95*	0.29	−0.67*	0.16
**Race/ethnicity**												
Hispanic	0.42	0.25	−0.79*	0.24	−0.36	0.26	−2.49*	0.28	0.04	0.27	0.51*	0.15
Non-Hispanic Black	−2.29*	0.59	−1.90*	0.58	0.99	0.60	−1.69	0.67	0.66	0.63	0.26	0.35
Non-Hispanic Other	−1.83*	0.45	−1.54*	0.44	0.93	0.47	−1.16	0.51	−0.16	0.48	0.42	0.27
Non-Hispanic White	0.00		0.00		0.00		0.00		0.00		0.00	
**Cancer site**												
Breast	3.57*	0.76	2.16*	0.75	1.19	0.81	3.55*	0.88	−2.89*	0.84	−0.94	0.46
GYN	4.00*	0.76	2.17*	0.75	3.71*	0.81	4.88*	0.86	−5.26*	0.83	−2.20*	0.45
GI	1.70*	0.54	0.97	0.53	2.02*	0.57	3.73*	0.61	−4.02*	0.60	−2.01*	0.32
Head and neck	1.32	0.67	0.47	0.66	1.43	0.71	2.08*	0.77	−1.48	0.74	−1.40*	0.40
Hematologic	3.34*	0.54	1.91*	0.52	2.89*	0.57	5.19*	0.61	−6.13*	0.59	−2.17*	0.32
Lung	2.31*	0.66	1.15	0.65	1.86*	0.70	3.05*	0.75	−3.34*	0.73	−1.43*	0.40
Prostate	0.00		0.00		0.00		0.00		0.00		0.00	
**Chemotherapy**	0.28	0.17	0.36	0.16	0.67*	0.19	1.92*	0.19	−1.63*	0.17	−0.80*	0.09
**Immunotherapy**	−0.09	0.33	0.07	0.31	1.48*	0.36	0.80	0.37	−0.92*	0.34	−0.26	0.19
**Hormonal therapy**	1.12*	0.30	0.87*	0.28	0.20	0.32	1.01*	0.33	−0.18	0.29	−0.60*	0.16
**Surgery**	1.44*	0.32	−0.26	0.30	8.39*	0.35	2.12*	0.35	−9.21*	0.33	−1.18*	0.18
**Radiation**	−0.21	0.47	−0.54	0.45	1.11	0.51	0.83	0.52	−0.55	0.46	−0.25	0.26
**Interaction**												
Time*breast	−0.02	0.02	−0.02	0.02	0.03	0.03	−0.04	0.03	0.00	0.03	0.01	0.01
Time*GYN	−0.01	0.03	0.01	0.03	−0.02	0.03	−0.03	0.03	0.01	0.03	0.02	0.01
Time*GI	0.01	0.02	0.01	0.02	−0.01	0.02	−0.04	0.02	0.02	0.02	0.02	0.01
Time*head and neck	−0.01	0.02	0.00	0.02	−0.03	0.02	−0.07*	0.03	0.04	0.02	0.03	0.01
Time*hematologic	−0.04	0.02	−0.01	0.02	−0.03	0.02	−0.07*	0.02	0.07*	0.02	0.03	0.01
Time*lung	0.01	0.02	0.01	0.02	−0.03	0.03	−0.03	0.03	0.00	0.03	0.01	0.01
Time*prostate	0.00		0.00		0.0		0.00		0.00		0.00	
**Random effects**												
Intercept variance	54.19*	2.86	54.27*	2.72	55.49*	3.23	64.53*	3.67	70.96*	3.47	18.53*	1.01
Time slope variance	0.01*	0.00	0.01*	0.00	0.01*	0.00	0.00	0.00	0.02*	0.00	0.00	0.00

Random intercepts and random slopes for time were included to account for inter-individual variability. Continuous predictors were grand-mean centered. Fixed-effect significance was determined using type III tests

Time: months since cancer diagnosis. Reference groups: cancer site: prostate; marital status: unpartnered; insurance status: uninsured; metastatic disease: no; chemotherapy: no; immunotherapy: no; hormonal therapy: no; surgery: no; radiation: no; interaction term: time*prostate

*GI*, gastrointestinal cancer; *GYN*, gynecological cancer; *HRQOL*, health-related quality of life; *SE*, standard error

*Statistically significant *p* values (Bonferroni-adjusted *p* <.0083)

### Anxiety

Anxiety scores varied significantly across patients at cancer diagnosis (random intercept = 54.19, *p* < 0.001). Having a partner (*B* = −1.06, *p* < 0.001) was associated with lower anxiety, while non-Hispanic Black patients (*B* = −2.29, *p* < 0.001) and those identifying as non-Hispanic Other (*B* = −1.83, *p* < 0.0001) reported lower anxiety than non-Hispanic Whites at cancer diagnosis. A greater number of comorbidities (*B* = 0.27, *p* < 0.001) was associated with increased anxiety. Receiving hormonal therapy (*B* = 1.12, *p* < 0.001) or surgery (*B* = 1.44, *p* < 0.001) at the time of assessment was associated with higher anxiety.

Overall, anxiety scores decreased over time (fixed III test *p* < 0.001) (Fig. 1a), with significant variation in individual rates of change (random-slope variance = 0.01, *p* = 0.002). However, the rate of change did not differ significantly by cancer type (interaction: *p* = 0.178). When examining cancer types, compared to prostate cancer, patients with all other cancer types except head and neck reported higher anxiety at diagnosis (all comparisons were significant at *p* < 0.008). Specifically, predicted anxiety scores remained significantly elevated for patients with GYN cancer than those with GI through 24 months, head and neck through 36 months, and prostate cancer through 60 months (Table S2). Conversely, patients with prostate cancer consistently reported lower anxiety scores than patients with breast, GYN, GI, hematologic, and lung cancers from diagnosis through the first 48 months (all comparisons were significant at *p* < 0.008).

### Depression

Depression scores varied significantly across patients at cancer diagnosis (random intercept = 54.27, *p* < 0.0001). Having a partner (*B* = −1.48, *p* < 0.001) was associated with lower depression, while Hispanic, non-Hispanic Black, and non-Hispanic Other patients reported lower depression than non-Hispanic Whites at cancer diagnosis (*B* = −1.90, *p* = 0.001). A greater number of comorbidities was associated with higher depression (*B* = 0.30, *p* < 0.001). Receiving hormonal therapy at the time of assessment was associated with higher depression levels (*B* = 0.87, *p* = 0.002).

Depression scores decreased over time overall (fixed III test *p* = 0.008), with significant variation in individual rates of change (random slope variance = 0.01, *p* = 0.04). However, the rate of change was similar across cancer types (interaction: *p* = 0.713). When examining cancer types, patients with breast, GYN, and hematologic cancers reported significantly higher depression at diagnosis than those with prostate cancer (all *p*’s < 0.005). Predicted depression scores indicated that the increased depressive burden for GYN and hematologic cancer patients persisted, remaining significantly higher than prostate cancer through the first 48 months (Table S2). Breast cancer patients exhibited significantly higher depression scores than those with prostate cancer early in the survivorship period (first 24 months) (all *p*’s < 0.008).

### Pain interference

At cancer diagnosis, pain interference scores varied significantly across patients (random intercept = 55.49, *p* < 0.001). Having a partner (*B* = −0.99, *p* < 0.001) was associated with lower pain interference. Higher pain interference scores at cancer diagnosis were significantly associated with older age (*B* = 0.04, p < 0.001) and comorbidities (*B* = 0.40, *p* < 0.001). Receiving chemotherapy (*B* = 0.67, *p* = 0.002) or immunotherapy (*B* = 1.48, p < 0.001) and undergoing surgery (*B* = 8.39, *p* < 0.001) at the time of assessment were significant predictors of higher pain interference scores.

Pain interference scores changed over time (fixed III test *p* = 0.007), with significant variation in individual rates of change (random slope variance = 0.01, *p* = 0.04). However, the rate of change was similar across cancer types (interaction: *p* = 0.289). When examining cancer types, compared to prostate cancer patients, patients with GYN, GI, hematologic, or lung cancer reported higher levels of pain interference at cancer diagnosis. Specifically, predicted mean scores showed that patients with GYN and hematologic cancers had the highest levels of pain interference across time, while prostate cancer patients reported the lowest pain interference across time (Table S2).

### Fatigue

At cancer diagnosis, fatigue scores varied significantly across the patients (random intercept = 64.53, *p* < 0.001). Having a partner (*B* = −1.07, *p* < 0.001) was associated with lower fatigue, while Hispanic patients reported lower fatigue than non-Hispanic Whites (*B* = −2.49, *p* < 0.001). A greater number of comorbidities at cancer diagnosis (*B* = 0.40, *p* < 0.001) and metastatic disease (*B* = 0.47,* p* < 0.001) were associated with higher fatigue. Receiving chemotherapy (*B* = 1.92, *p* < 0.001) and undergoing surgery (*B* = 2.12, *p* < 0.001) at the time of assessment were associated with higher fatigue.

Overall, fatigue scores decreased over time (fixed III test *p* < 0.001), with no significant variation in individual rates of change (random slope variance = 0.00, *p* = 0.478). When examining cancer types, patients with prostate cancer reported the lowest fatigue levels at cancer diagnosis. Furthermore, the rate of change was different across cancer types. Compared to patients with prostate cancer, those with head and neck (*B* = −0.07, *p* = 0.006) and hematologic malignancies (*B* = −0.07, *p* = 0.001) reported an improvement in fatigue over time. Predicted fatigue scores showed that patients with GYN and hematologic malignancies maintained significantly higher fatigue than prostate cancer patients through the first 48 months (Fig. 1d; Table S2). Patients with head and neck cancer showed significantly lower predicted fatigue scores than those with GYN and hematologic cancer from diagnosis through 60 months (all comparisons were significant at *p* < 0.008).

### Physical function

Physical function scores varied significantly across patients at cancer diagnosis (random intercept = 70.96, *p* < 0.001). Older age (*B* = −0.22), a higher number of comorbidities (*B* = −0.63), and metastatic disease (*B* = −0.95) were associated with significantly lower physical function scores at cancer diagnosis. Receiving chemotherapy (*B* = −1.63), immunotherapy (*B* = −0.92), and undergoing surgery at the time of assessment (*B* = −9.21) were associated with significantly lower physical function scores (all *p*’s ≤ 0.001). Conversely, having a partner (*B* = 1.05, *p* < 0.001) was associated with significantly higher physical function scores.

Overall, physical function scores improved over time (fixed III test *p* < 0.001), with significant variation in individual rates of change (random slope variance = 0.02, *p* < 0.001). When examining cancer types, compared to patients with prostate cancer, all other cancer types except head and neck reported lower physical function at cancer diagnosis. Patients with hematologic malignancies faced the greatest burden at cancer diagnosis; however, they showed significant greater improvements in physical functioning over time (*B* = 0.07, *p* = 0.0003). Predicted mean scores indicated that patients with prostate cancer exhibited significantly higher physical function scores through 60 months compared to those with breast, GI, hematologic, and lung cancer (Table S2). Similarly, patients with head and neck cancer had significantly higher physical function scores compared to those with breast, GI, hematologic, and lung cancer from 12 months onward. In contrast, patients with GYN cancer consistently exhibited lower physical function scores across time than patients with prostate and head and neck cancer (all comparisons were significant at *p* < 0.008).

### HRQOL

HRQOL scores varied significantly across patients at cancer diagnosis (random intercept = 18.53, *p* < 0.001). A higher number of comorbidities (*B* = −0.28) and metastatic disease (*B* = −0.67) were associated with lower HRQOL at cancer diagnosis. Receiving chemotherapy (*B* = −0.80), hormonal therapy (*B* = −0.60), or surgery (*B* = −1.18) at the time of assessment was associated with lower HRQOL scores (*p*’s < 0.001). Having a partner (*B* = 0.72, *p* < 0.001) was associated with higher HRQOL scores at cancer diagnosis.

Overall, HRQOL scores significantly change over time (fixed III test *p* < 0.001). However, the rate of change was similar across cancer types (interaction: *p* = 0.170). When examining cancer types, compared to prostate cancer, all other cancer types except breast reported lower HRQOL at cancer diagnosis (all *p*’s < 0.001). Although not statistically significant, patients with head and neck cancer showed improvements in HRQOL over time compared to those with prostate cancer (*B* = 0.03, *p* = 0.026) (Fig. 1f). Prostate cancer patients had significantly higher predicted HRQOL scores than those with GYN, GI, hematologic, and lung cancer through the first 36 months (Table S2). Similarly, patients with head and neck cancer had significantly higher HRQOL scores compared to those with hematologic malignancies from diagnosis through the first 48 months. By 60 months, there are no differences between cancer types, suggesting a long-term stabilization in HRQOL.

## Discussion

This study provides a comprehensive analysis of longitudinal PROs among a diverse cohort of diverse cancer survivors receiving ambulatory oncology care. Across multiple PRO domains including anxiety, depression, pain interference, fatigue, physical function, and HRQOL, we identified distinct patterns of overall symptom burden that persist over time across cancer types. Rather than reflecting large differences in rate of change, these patterns were primarily characterized by stable between-cancer differences in symptom levels, with some notable exceptions. Patients with GYN cancer consistently reported the highest symptom burden across domains, whereas patients with prostate cancer reported a low-burden symptom profile with more favorable PRO scores. Patients with head and neck cancer showed improvement over time in fatigue, while patients with hematologic malignancies improved in physical functioning over time compared to patients with prostate cancer. In addition, social and clinical factors, including marital status, comorbidity burden, and treatment modalities, were associated with PROs, highlighting the multifactorial nature of survivorship symptom experiences.

Our findings align with existing literature that underscores the heterogeneity in PROs across cancer populations. The relatively low symptom burden observed in patients with prostate cancer has been previously reported, reflecting the generally favorable prognosis and comparatively lower treatment-related morbidity in many patients [27, 28]. Conversely, patients with GYN cancer exhibited the high-burden symptom profile, particularly in anxiety, pain interference, fatigue, and physical function. These findings are consistent with studies suggesting cumulative psychosocial and physical impact of multimodal treatments and treatment-related effect on reproductive and sexual health [29, 30]. Beyond clinical factors, cultural or social factors may influence how patients report symptoms. For instance, men adhering to traditional masculine norms may hesitate to discuss symptoms due to fear of vulnerability or weakness openly [31]. Additionally, certain symptoms, such as mental health problems, carry a stigma, leading some patients to underreport or avoid discussing them [32, 33]. Understanding each patient’s unique circumstances is essential for effective cancer care.

Patients with hematologic malignancies demonstrated a more complex symptom profile, characterized by relatively high overall burden in domains such as depression and pain interference, alongside improvement in physical function over time. This mixed pattern is consistent with prior survivorship literature, which suggests that patients with hematology malignancies often experience substantial symptom burden related to systemic disease and intensive treatments, yet, may show functional recovery during periods of remission or treatment completion [34]. Importantly, treatment exposures, including chemotherapy, immunotherapy, hormonal therapy, and surgery, were strong and consistent predictors of worse PROs across cancer types, highlighting the central role of active treatment in shaping symptom experiences. By explicitly accounting for cancer type, disease stage, treatment status, and time since diagnosis in our models, we aimed to disentangle cancer-specific patterns from treatment-related effects. These findings reinforce the need to consider both disease and treatment context when interpreting PRO trajectories and designing survivorship care strategies.

The observed variability in PRO trajectories across cancer type suggests that a one-size-fits-all approach to symptom management in oncology is insufficient. Cancer types characterized by persistently low symptom burden, such as patients with prostate, and in several domains, head and neck cancers, may be adequately managed with standard follow-up and routine symptom monitoring. In contrast, the consistently elevated symptom burden observed among patients with GYN cancer highlights the need for more intensive and tailored interventions. Multidisciplinary survivorship clinics that integrate oncology, psychosocial support, palliative care, and rehabilitation services may be particularly beneficial for these high-risk groups. Leveraging longitudinal PRO data through EHR-integrated systems, such as those using PROMIS-based thresholds, can facilitate timely symptom identification and clinical response. Our findings suggest that alert thresholds and response protocols may need to be refined by cancer type to enhance clinical relevance and prioritize patients at greatest risk for distress and functional decline.

The significant associations between marital status and more favorable PRO scores across all domains underscore the potential role of social support in mitigating symptom burden. Interventions to strengthen social networks, particularly for unpartnered patients, could enhance their overall well-being [35, 36]. For instance, connecting cancer patients with others who have similar experiences can provide a sense of community and emotional support. Furthermore, online support groups can offer a convenient way for patients to connect with others, especially those in rural or isolated areas. We also found that comorbidities were associated with worse PROs. Managing multiple health conditions can be overwhelming and contribute to increased anxiety, depression, and stress. Additionally, comorbidities can interact with cancer treatments, leading to adverse side effects or complications. Our findings emphasize the importance of managing comorbidities in cancer survivors to improve their quality of life.

This study has several strengths, including a large, diverse sample, use of validated EHR-integrated PRO system, and consistent measurement across time points. The longitudinal design enabled characterization of PRO trajectories, providing insights that are not possible with cross-sectional studies. Notably, by focusing exclusively on patients within 5 years of cancer diagnosis, this study minimizes the confounding effects of “long-term” survivorship. This ensures that the observed trajectories reflect the transition from acute treatment to early stabilization. Furthermore, this study leveraged real-world data from routine clinical care, unlike many prior studies based on clinical trials or registries, and examined multiple PRO domains across diverse cancer types, offering a comprehensive view of patient experiences. However, there are limitations to consider. The study’s observational nature precludes causal inferences, and the variability in assessment intervals may introduce bias in the trajectory analysis. Additionally, inclusion of only seven cancer types and conduct at a single institution limit generalizability. Furthermore, PRO trajectories may differ among patients outside ambulatory oncology settings, such as hospitalized or hospice patients, who often experience higher and more dynamic symptom burden. In addition, survivorship bias should be considered when interpreting these findings. Follow-up duration and the number of completed PRO assessments may differ by cancer type due to variation in disease trajectory, treatment intensity, and mortality risk. As a result, patients with more aggressive disease or poorer prognosis may be underrepresented at later time points, potentially leading to an overestimation of symptom improvement over time in some groups. Optional participation in the MWC questionnaire and attrition over time introduce the potential for self-selection and missing data bias, further limiting generalizability. Although our modeling captured average trends, individual symptom trajectory may vary substantially. For example, hematologic malignancies (leukemia, lymphoma, and others) were analyzed as a single group, which may obscure disease-specific differences in symptom trajectories. However, this pragmatic approach is consistent with prior PRO literature and reflects shared survivorship symptom burden and treatment patterns in real-world clinical care [37, 38]. Disease stage and treatment type were included as covariates to mitigate heterogeneity; however, while treatment was modeled as a time-varying covariate to capture active clinical management, other clinical factors such as cancer stage and comorbidities were treated as time-invariant based on baseline records. We acknowledge that changes in these clinical statuses over time could influence symptom trajectories, and the lack of time-varying data for these variables represents an additional limitation of this study.

Future research should develop targeted interventions for cancer types with higher symptom burden and explore the underlying biological, psychological, and social mechanisms driving the differences. Incorporating patient preferences and values into care planning will be crucial for enhancing patient-centered oncology care.

## Conclusions

This study highlights the meaningful differences in overall symptom burden and HRQOL across cancer types in ambulatory care. These findings highlight the need for personalized and targeted interventions. Identifying patients at higher risk for poor PRO outcomes enables tailored support, ultimately improving their quality of life and treatment experience.

## Supplementary Information

Below is the link to the electronic supplementary material.

ESM1 (DOCX 46.0 KB)

## Data Availability

The datasets generated during and/or analysed during the current study are available from the corresponding author on reasonable request.
