# Supplementary material for: Trajectories of patient-reported outcomes among diverse cancer patients in ambulatory oncology clinics
Source: J Cancer Surviv. 2026 Jun 4;20(Suppl 2):2165–77. doi: 10.1007/s11764-026-02050-0 (PMC13744504; doi:10.1007/s11764-026-02050-0)
Supplement: Supplementary file 1 [file 11764_2026_2050_MOESM1_ESM.docx]

**Supplementary Information**

**Manuscript Title:**

**Trajectories of Patient-Reported Outcomes among Diverse Cancer Patients in Ambulatory Oncology Clinics.**

**Authors:** Akina Natori, Blanca Silvia Noriega Esquives, Vandana D. Sookdeo, Rui Gong, Jessica MacIntyre, Carmen Calfa, Tracy E. Crane, Matthew Schlumbrecht, Frank J. Penedo

Journal: Journal of Cancer Survivorship

Corresponding Author: Frank J. Penedo, PhD

Professor of Psychology and Medicine, Miller School of Medicine, University of Miami

Associate Director for Cancer Survivorship and Translational Behavioral Sciences

Director of Cancer Survivorship and Supportive Care, Sylvester Comprehensive Cancer Center

1120 NW 14th Street, Miami, FL 33136

Email: fpenedo@miami.edu

Table S1. Differences between only one and multiple assessment completers.

|  | **Only One Assessment (n=2,258)** | | **Multiple Assessments**  **(n=4,681)** | | ***p*** |
| --- | --- | --- | --- | --- | --- |
|  | **Mean or N** | **SD or %** | **Mean or N** | **SD or %** |  |
| Age | 63.1 | 13.4 | 63.4 | 12.9 | 0.4669 |
| Female | 904 | 40 | 2101 | 44.9 | **0.0001** |
| Partnered | 1378 | 61 | 2968 | 63.4 | 0.3085 |
| Insured | 2199 | 97.4 | 4586 | 98 | 0.0955 |
| Race/Ethnicity |  |  |  |  | **<.0001** |
| Non-Hispanic White | 842 | 37.3 | 1923 | 41.1 |  |
| Non-Hispanic Black | 0 | 0 | 224 | 4.79 |  |
| Non-Hispanic Other | 307 | 13.6 | 284 | 6.07 |  |
| Hispanic | 986 | 43.7 | 2029 | 43.3 |  |
| Charlson Comorbidity Index | 5.22 | 3.56 | 6.12 | 3.61 | **<.0001** |
| Cancer Site |  |  |  |  | **<.0001** |
| Breast | 137 | 6.07 | 381 | 8.14 |  |
| GYN | 125 | 5.54 | 300 | 6.41 |  |
| GI | 443 | 19.6 | 975 | 20.8 |  |
| Head and Neck | 214 | 9.48 | 482 | 10.3 |  |
| Hematologic | 579 | 25.6 | 1223 | 26.1 |  |
| Lung | 201 | 8.9 | 451 | 9.63 |  |
| Prostate | 559 | 24.8 | 869 | 18.6 |  |
| Months since diagnosis | 18.8 | 17.9 | 26.7 | 18.5 | **<.0001** |
| Metastatic disease | 1092 | 48.4 | 1896 | 40.5 | **<.0001** |
| Treatment Type |  |  |  |  |  |
| Chemotherapy | 431 | 19.1 | 1261 | 26.9 | **<.0001** |
| Immunotherapy | 78 | 3.45 | 243 | 5.19 | **0.0013** |
| Hormonal Therapy | 101 | 4.47 | 333 | 7.11 | **<.0001** |
| Surgery | 206 | 9.12 | 370 | 7.9 | 0.0847 |
| Radiation | 30 | 1.33 | 93 | 1.99 | 0.0516 |
| Patient Reported Outcome |  |  |  |  |  |
| Anxiety | 50.5 | 10.6 | 49.9 | 10.7 | 0.0394 |
| Depression | 46.3 | 10 | 46.3 | 10.1 | 0.8331 |
| Pain Interference | 51.6 | 11 | 50.6 | 10.9 | **0.0014** |
| Fatigue | 48.3 | 11.8 | 48.4 | 11.8 | 0.6581 |
| Physical Function | 44.8 | 11.9 | 45.5 | 11.5 | 0.0324 |
| HRQOL | 19.3 | 6.11 | 19.4 | 5.9 | 0.5374 |

GI; gastrointestinal cancer, GYN; gynecological cancer, HRQOL; health-related quality of life, SD; standard deviation. Statistically significant p values (Bonferroni-adjusted p<.0083).

Table S2. Predicted Patient-Reported Outcomes Scores Across Time by Cancer Type

|  | **Breast** | | **GYN** | | **GI** | | **Head and Neck** | | **Hematologic** | | **Lung** | | **Prostate** | |
| --- | --- | --- | --- | --- | --- | --- | --- | --- | --- | --- | --- | --- | --- | --- |
| **Time** | **Predicted Mean** | **SE** | **Predicted Mean** | **SE** | **Predicted Mean** | **SE** | **Predicted Mean** | **SE** | **Predicted Mean** | **SE** | **Predicted Mean** | **SE** | **Predicted Mean** | **SE** |
| **PROMIS Anxiety** | | | | | | | | | | | | | | |
| **0** | 53.60 | 0.82 | 54.03 | 0.83 | 51.73 | 0.63 | 51.35 | 0.75 | 53.37 | 0.65 | 52.34 | 0.72 | 50.03 | 0.63 |
| **6** | 53.28 | 0.75 | 53.73 | 0.76 | 51.55 | 0.61 | 51.10 | 0.70 | 52.92 | 0.63 | 52.17 | 0.67 | 49.82 | 0.60 |
| **12** | 52.97 | 0.70 | 53.43 | 0.72 | 51.37 | 0.59 | 50.84 | 0.66 | 52.47 | 0.61 | 52.01 | 0.65 | 49.61 | 0.59 |
| **24** | 52.34 | 0.67 | 52.84 | 0.71 | 51.01 | 0.60 | 50.33 | 0.64 | 51.57 | 0.60 | 51.67 | 0.65 | 49.18 | 0.59 |
| **36** | 51.71 | 0.72 | 52.24 | 0.79 | 50.65 | 0.65 | 49.82 | 0.69 | 50.67 | 0.62 | 51.34 | 0.75 | 48.75 | 0.64 |
| **48** | 51.08 | 0.85 | 51.64 | 0.96 | 50.29 | 0.73 | 49.30 | 0.80 | 49.77 | 0.66 | 51.01 | 0.89 | 48.33 | 0.72 |
| **60** | 50.45 | 1.02 | 51.05 | 1.16 | 49.93 | 0.84 | 48.79 | 0.95 | 48.88 | 0.74 | 50.67 | 1.08 | 47.90 | 0.84 |
| **PROMIS Depression** | | | | | | | | | | | | | | |
| **0** | 48.29 | 0.81 | 48.30 | 0.81 | 47.10 | 0.62 | 46.60 | 0.74 | 48.04 | 0.63 | 47.27 | 0.71 | 46.13 | 0.62 |
| **6** | 48.07 | 0.74 | 48.25 | 0.75 | 47.07 | 0.59 | 46.47 | 0.69 | 47.87 | 0.61 | 47.25 | 0.66 | 46.02 | 0.59 |
| **12** | 47.84 | 0.69 | 48.21 | 0.71 | 47.04 | 0.58 | 46.33 | 0.65 | 47.69 | 0.59 | 47.23 | 0.63 | 45.91 | 0.57 |
| **24** | 47.40 | 0.66 | 48.12 | 0.69 | 46.98 | 0.58 | 46.06 | 0.63 | 47.34 | 0.58 | 47.19 | 0.64 | 45.69 | 0.58 |
| **36** | 46.95 | 0.70 | 48.03 | 0.77 | 46.92 | 0.63 | 45.79 | 0.67 | 46.99 | 0.60 | 47.15 | 0.72 | 45.47 | 0.62 |
| **48** | 46.51 | 0.82 | 47.94 | 0.91 | 46.86 | 0.70 | 45.52 | 0.77 | 46.64 | 0.64 | 47.10 | 0.86 | 45.25 | 0.70 |
| **60** | 46.06 | 0.98 | 47.85 | 1.10 | 46.80 | 0.81 | 45.25 | 0.91 | 46.28 | 0.72 | 47.06 | 1.03 | 45.03 | 0.80 |
| **PROMIS Pain Interference** | | | | | | | | | | | | | | |
| **0** | 57.11 | 0.87 | 59.64 | 0.88 | 57.95 | 0.68 | 57.35 | 0.80 | 58.82 | 0.69 | 57.79 | 0.77 | 55.93 | 0.67 |
| **6** | 57.26 | 0.80 | 59.49 | 0.81 | 57.83 | 0.65 | 57.13 | 0.75 | 58.62 | 0.67 | 57.58 | 0.72 | 55.89 | 0.64 |
| **12** | 57.40 | 0.75 | 59.33 | 0.77 | 57.70 | 0.63 | 56.90 | 0.71 | 58.43 | 0.65 | 57.38 | 0.69 | 55.84 | 0.63 |
| **24** | 57.68 | 0.71 | 59.03 | 0.75 | 57.44 | 0.64 | 56.44 | 0.68 | 58.03 | 0.63 | 56.97 | 0.69 | 55.77 | 0.63 |
| **36** | 57.97 | 0.76 | 58.72 | 0.83 | 57.19 | 0.68 | 55.98 | 0.73 | 58.64 | 0.65 | 56.56 | 0.78 | 55.69 | 0.67 |
| **48** | 58.25 | 0.88 | 58.42 | 0.99 | 56.94 | 0.77 | 55.53 | 0.84 | 57.25 | 0.70 | 56.15 | 0.93 | 55.61 | 0.76 |
| **60** | 58.54 | 1.06 | 58.11 | 1.20 | 56.69 | 0.88 | 55.07 | 0.99 | 56.86 | 0.78 | 55.74 | 1.12 | 55.53 | 0.87 |
| **PROMIS Fatigue** | | | | | | | | | | | | | | |
| **0** | 52.82 | 0.94 | 54.15 | 0.94 | 53.00 | 0.72 | 51.35 | 0.86 | 54.46 | 0.74 | 52.32 | 0.82 | 49.27 | 0.72 |
| **6** | 52.63 | 0.86 | 54.02 | 0.87 | 52.81 | 0.69 | 50.99 | 0.80 | 54.11 | 0.71 | 52.23 | 0.77 | 49.33 | 0.69 |
| **12** | 52.44 | 0.81 | 53.88 | 0.82 | 52.61 | 0.67 | 50.63 | 0.76 | 53.76 | 0.69 | 52.13 | 0.74 | 49.39 | 0.67 |
| **24** | 52.05 | 0.77 | 53.61 | 0.80 | 52.22 | 0.68 | 49.90 | 0.73 | 53.07 | 0.68 | 51.93 | 0.74 | 49.51 | 0.67 |
| **36** | 51.66 | 0.82 | 53.34 | 0.89 | 51.83 | 0.73 | 49.17 | 0.78 | 52.37 | 0.70 | 51.73 | 0.84 | 49.63 | 0.72 |
| **48** | 51.28 | 0.96 | 53.07 | 1.06 | 51.43 | 0.83 | 48.45 | 0.90 | 51.68 | 0.75 | 51.53 | 1.00 | 49.75 | 0.81 |
| **60** | 50.89 | 1.15 | 52.80 | 1.28 | 51.04 | 0.95 | 47.72 | 1.06 | 50.98 | 0.84 | 51.34 | 1.20 | 49.86 | 0.93 |
| **PROMIS Physical Function** | | | | | | | | | | | | | | |
| **0** | 38.85 | 0.89 | 36.47 | 0.90 | 37.71 | 0.68 | 40.25 | 0.81 | 35.60 | 0.69 | 38.39 | 0.78 | 41.73 | 0.68 |
| **6** | 38.95 | 0.82 | 36.67 | 0.82 | 37.98 | 0.65 | 40.59 | 0.76 | 35.16 | 0.66 | 38.49 | 0.73 | 41.86 | 0.65 |
| **12** | 39.05 | 0.76 | 36.86 | 0.77 | 38.25 | 0.63 | 40.94 | 0.71 | 36.71 | 0.64 | 38.58 | 0.69 | 41.98 | 0.63 |
| **24** | 39.25 | 0.71 | 37.25 | 0.75 | 38.79 | 0.63 | 41.62 | 0.68 | 37.81 | 0.62 | 38.77 | 0.70 | 42.23 | 0.63 |
| **36** | 39.46 | 0.76 | 37.64 | 0.84 | 39.34 | 0.68 | 42.31 | 0.73 | 38.91 | 0.64 | 38.95 | 0.79 | 42.47 | 0.67 |
| **48** | 39.66 | 0.90 | 38.04 | 1.01 | 39.88 | 0.77 | 42.99 | 0.84 | 40.02 | 0.70 | 39.14 | 0.94 | 42.72 | 0.76 |
| **60** | 39.86 | 1.08 | 38.43 | 1.23 | 40.42 | 0.89 | 43.68 | 1.00 | 41.12 | 0.78 | 39.33 | 1.13 | 42.96 | 0.88 |
| **FACT-G7 HRQOL** | | | | | | | | | | | | | | |
| **0** | 17.64 | 0.49 | 16.38 | 0.49 | 16.57 | 0.37 | 17.18 | 0.45 | 16.41 | 0.38 | 17.15 | 0.43 | 18.58 | 0.38 |
| **6** | 17.72 | 0.45 | 16.56 | 0.45 | 16.74 | 0.36 | 17.40 | 0.42 | 16.61 | 0.37 | 17.25 | 0.40 | 18.63 | 0.36 |
| **12** | 17.80 | 0.42 | 16.73 | 0.42 | 16.92 | 0.35 | 17.62 | 0.39 | 16.82 | 0.36 | 17.35 | 0.38 | 18.67 | 0.35 |
| **24** | 17.97 | 0.39 | 17.09 | 0.41 | 17.27 | 0.35 | 18.07 | 0.38 | 17.22 | 0.35 | 17.56 | 0.38 | 18.77 | 0.35 |
| **36** | 18.13 | 0.42 | 17.45 | 0.46 | 17.62 | 0.38 | 18.52 | 0.40 | 17.63 | 0.36 | 17.76 | 0.43 | 18.87 | 0.37 |
| **48** | 18.29 | 0.49 | 17.80 | 0.54 | 17.97 | 0.43 | 18.96 | 0.46 | 18.03 | 0.39 | 17.97 | 0.51 | 18.97 | 0.42 |
| **60** | 18.45 | 0.59 | 18.16 | 0.65 | 18.32 | 0.49 | 19.41 | 0.54 | 18.44 | 0.43 | 18.17 | 0.62 | 19.07 | 0.48 |

GI; gastrointestinal cancer, GYN; gynecological cancer, HRQOL; health-related quality of life, SE; standard error. Time: months since cancer diagnosis.

Statistically significant *p* values (Bonferroni-adjusted p<.0083).
